# Are YouTube Videos a Useful and Reliable Source of Information for Patients with Temporomandibular Joint Disorders?

**DOI:** 10.3390/jcm12030817

**Published:** 2023-01-19

**Authors:** Luigi Angelo Vaira, Silvia Sergnese, Giovanni Salzano, Fabio Maglitto, Antonio Arena, Emanuele Carraturo, Vincenzo Abbate, Umberto Committeri, Valentino Vellone, Andrea Biglio, Jérome R. Lechien, Giacomo De Riu

**Affiliations:** 1 Maxillofacial Surgery Operative Unit, Department of Medicine, Surgery and Pharmacy, University of Sassari, 07100 Sassari, Italy; 2 Biomedical Science Department, PhD School of Biomedical Science, University of Sassari, 07100 Sassari, Italy; 3 Maxillofacial Surgery Operative Unit, University Hospital of Naples “Federico II”, 80131 Naples, Italy; 4 Department of Maxillofacial Surgery, Santa Maria Hospital, 05100 Terni, Italy; 5 Department of Anatomy and Experimental Oncology, Mons School of Medicine, UMONS, Research Institute for Health Sciences and Technology, University of Mons (UMons), 7000 Mons, Belgium; 6 Department of Otolaryngology-Head Neck Surgery, Elsan Polyclinic of Poitiers, 86000 Poitiers, France

**Keywords:** YouTube, temporomandibular joint disorder, counseling, internal derangement, temporomandibular joint, counseling, maxillofacial surgery

## Abstract

Counseling is considered a first-line conservative therapy with respect to temporomandibular joint disorders (TMJD). Nowadays, 50 to 80% of patients acquire health information from the internet before turning to professionals. The purpose of this study has been to investigate the quality of information about TMJD that patients can obtain from YouTube. A YouTube.com search was conducted using the terms “temporomandibular joint disorder”; “limited movement of the mandible”; and “mandibular joint pain”. The videos identified were assessed independently by two panels of three professional and lay reviewers with HONcode, modified DISCERN (MD) and the global quality scale (GQS). A total of 106 videos were included. The professional reviewers reported a mean HONcode score of 4.148 ± 1.314 and a mean MD score of 2.519 ± 1.267, testifying to a modest general quality of the videos. The mean GQS score was 2.987 ± 1.012 for the professional and 3.469 ± 0.891 for the lay reviewers (*p* < 0.001). The correlations between the ratings were significant between the reviewers within the same group but not between the two groups. The presence of animations significantly influenced the GQS score expressed by the lay reviewers (*p* = 0.011) but not that of the professionals (*p* = 0.640). The quality of the information on TMJD on YouTube is generally of poor quality. Healthcare systems and professionals should be prepared to correct misinformation and build trusting relationships with patients which are based on quality counseling. Similarly, academic institutions should produce quality content that leads patients with TMJD toward a correct diagnostic-therapeutic process.

## 1. Introduction

Temporomandibular disorders include a broad spectrum of pathologies affecting the temporomandibular joint and masticatory muscles. The prevalence of temporomandibular joint disorders (TMJD) is very high. Indeed, it is estimated that at least 40% of the population have at least one sign or symptom and that about 5% require assistance for this disabling disease [1,2]. Up to now, numerous treatments have been proposed for TMJD, which include drug therapies [3,4], physiotherapy [5], occlusal splints [6,7], arthrocentesis [8,9,10], arthroscopy [11] and open surgery [12].

Like these measures, counseling is considered a first-line conservative therapy [13,14,15,16]. The effectiveness of counseling in reducing symptoms of TMJD has been demonstrated by numerous authors and is comparable to that of intraoral devices [13,14,15,16,17,18]. Despite the high prevalence in the general population, knowledge about TMJD is often deficient, even among dentists and head and neck surgeons [19,20]. Furthermore, counseling, understood as the transmission of knowledge, is essential to raise patient awareness and achieve a correct diagnosis based on symptoms which, especially in the earliest stages, are often vague and do not lead the patient to seek treatment.

Nowadays, 50 to 80% of patients acquire health information from the internet before turning to professionals [21,22]. In particular, the video-sharing platform YouTube is the second most visited site in the world and one of the most frequently consulted sources by the lay public looking for health information [23]. However, the information that can be acquired on this platform is not peer-reviewed and often of poor quality, which could put the patient’s health at risk [24,25]. Moreover, these data can influence patient expectations and, consequently, impact on the doctor–patient relationship, posing medico-legal issues [26]. For this reason, in recent years, many articles have been published regarding the quality of information available on YouTube on numerous diseases and treatments of the head and neck district such as third molar extractions [27], orthognathic surgery [28], implantology [29], esthetic medicine and surgery [30] and head and neck cancers [31].

To the best of our knowledge, there have been no studies investigating the quality of information about TMJD on YouTube. Therefore, the aim of this study has been an attempt to fill this gap.

## 2. Materials and Methods

A YouTube.com search was performed on 16 July 2022 using the terms “temporomandibular joint disorder”, “limited movement of the mandible” and “mandibular joint pain”. These criteria were established based on the most searched terms by TMJD patients on Google trends. The search was carried out with a new account, after the navigation history and cookies had been deleted, in order not to affect the order in which the videos were presented. For each of the keywords, the videos were sorted by relevance and only the first three pages of results were considered for the purposes of the study. The inclusion was limited to the first 3 pages because only 17% of users view videos beyond this limit [32]. The videos were then preliminarily and independently evaluated by two researchers (L.A.V. and S.S.) who rejected those that presented one of the following exclusion criteria: duplication, advertisements, personal experiences, non-human, non-English or non-surgical data, and videos with fewer than 10,000 views.

For all the videos included, some general parameters were recorded: the presence of animations, the number of views, the duration, the date of publication, the continent of origin, the YouTube category and the total number of likes, dislikes and comments.

The list of videos with their respective links was then provided to the evaluation groups. The first group included three maxillofacial surgeons with a long experience in the treatment of TMJD and a C2 English language level. The second group included three laymen, native English speakers, who had never worked in healthcare and had never suffered from a TMJD or dealt with patients with a TMJD. The groups were matched for age and gender. The individuals of the professional group viewed all the videos and scored them on three different well-validated health information quality scales [33]: the Health on the Net Foundation code of conduct tool (HONcode), the Global Quality Scale (GQS) and the modified DISCERN (MD) scale. Individuals in the lay group rated the videos with the Global Quality Scale alone.

### 2.1. The Health on the Net Foundation Code of Conduct Tool

HONcode [Table 1] is a widely used tool for the evaluation of the reliability of the healthcare information reported on websites [34,35,36]. It investigates eight information characteristics (e.g., authoritativeness, complementarity, privacy, attribution, justifiability, transparency, financial disclosure and advertising policy) by applying a dichotomous assessment, namely 0 points if absent or 1 point if present. The score can therefore vary from 0 (no credibility of the information) to 8 (maximum credibility of the information).

### 2.2. Modified DISCERN

MD (Table 2) represents a modification of the original DISCERN scale to accommodate its use for evaluating videos [37,38,39]. It evaluates five video characteristics as present (score 1) or absent (score 0): clarity, reliability, bias/balance, the provision of additional information sources, and the recognition of areas of uncertainty. The score can therefore vary from 0 (poor quality) to 5 (excellent quality).

### 2.3. Global Quality Scale

The GQS (see Table 3), introduced and validated by Bernard et al. [37], evaluates the quality and information flow of the videos and consequently their usefulness for the patient. The scale assigns a score that varies from 1 (poor quality, a poor flow of the video, a lack of usefulness for the patient) to 5 (excellent quality and flow, significant usefulness for the patient)

### 2.4. Statistical Analysis

The statistical analyses were performed using Jamovi version 2.3.18.0, which is a freeware and open statistical software available online at www.jamovi.org (accessed on 20 November 2022) [40]. The categorical variables are reported in numerals and percentages of the total. Descriptive statistics for the quantitative variables are given as the median (interquartile range (IQR)). The agreement between the reviewers within the groups was analyzed using the interclass correlation coefficient with the Cronbach alpha. For each video, the medians of the three GCS scores of each group were compared with the Wilcoxon signed-rank test to assess the significance of intergroup differences. The Pearson’s correlation coefficient was used to evaluate the correlations between the median scores and the viewers’ interaction index and viewing rate [41].

$$Interaction\;index=\frac{N{}^{\circ}\;of\;likes+N{}^{\circ}\;of\;comments\;}{N{}^{\circ}of\;views}\times 100\%$$

$$Viewing\;rate=\frac{N{}^{\circ}\;of\;views}{N{}^{\circ}\;of\;days\;since\;upload}\times 100\%$$

The level of statistical significance was set at *p* < 0.05 with a 95% confidence interval.

## 3. Results

The keywords search on YouTube returned 316 videos. Of them, 210 were excluded for the following reasons: fewer than 10,000 views (89 videos), duplicates (11 videos), not relevant (103 videos), not in English (4 videos), non-human (2 videos) or no sound (1 video). Overall, 106 videos were then included and evaluated by the reviewers: 78 videos originated from North America, 12 from Europe, 10 from Asia and 6 from Oceania. The videos had an average duration of 459 ± 456 s (range 32–3710 s) and an average number of views of 222,160 ± 437,421 views (range 10,102–3,133,299 views). The mean viewing rate and interaction index were 142 ± 259 (range 1.26–2009) and 1.76 ± 1.39 (range 0.11–7.85), respectively. In addition, 31.1% of the videos featured animations.

### 3.1. The HONcode Results

The three professional reviewers reported an average HONcode score of 4.148 ± 1.314. In particular, the videos analyzed complied with the principles of attribution, financial disclosure and advertising policy only in 31.1%, 11.3% and 14.1% of the cases, respectively. The three reviewers agreed on the conformity with the justifiability and transparency criteria in 50.9 and 51.8% of the cases. The three principles most frequently respected were privacy (95.2%), complementarity (89.6%) and authority (64.1%).

**Figure 1:**
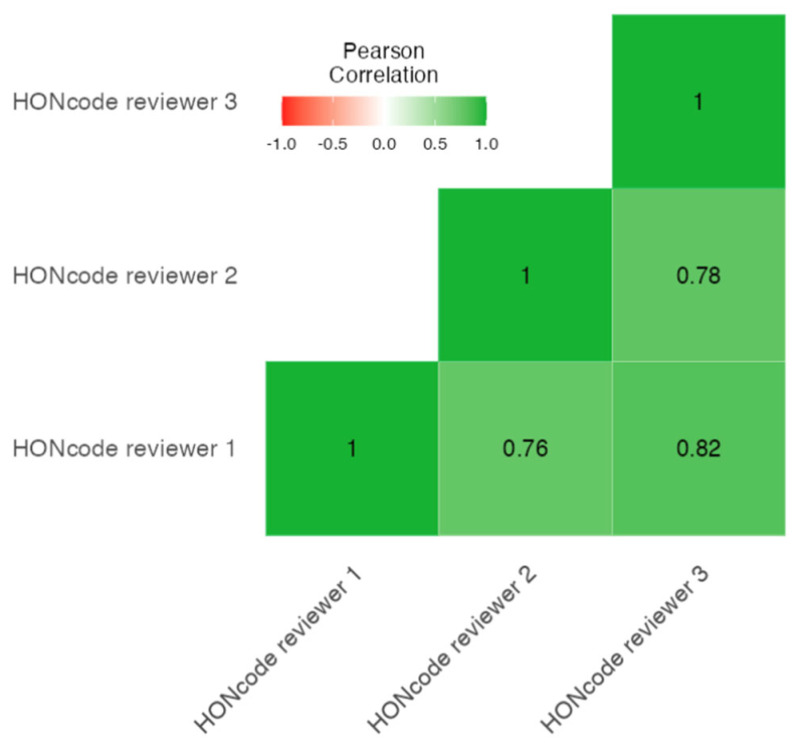
Heat map of the correlation between the HONcode scores of the three reviewers.

The evaluations of the three reviewers showed a strong and significant correlation (Cronbach α 0.913; *p* < 0.001) [Figure 1].

The median HONcode score of the three reviewers did not present any significant correlations with the number of views (r_p_ = −0.014, *p* = 0.887), duration (r_p_ = 0.152, *p* = 0.119), interaction index (r_p_ = 0.109, *p* = 0.268), viewing rate (r_p_ = 0.025, *p* = 0.801), continent of origin (χ^2^ = 6.30, *p* = 0.098) or presence of animations (F = 0.346, *p* = 0.559).

### 3.2. The Modified DISCERN Results

**Figure 2:**
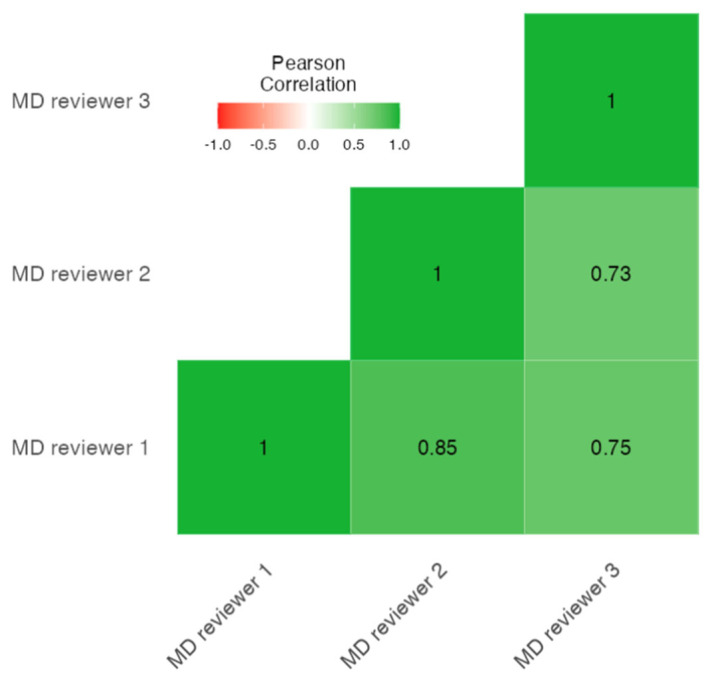
Heat map of the correlation between the MD scores of the three reviewers.

The mean MD score was 2.519 ± 1.267 testifying to a modest general quality of the videos. The reviewers agreed that all five of the evaluated characteristics were present in only two videos. In particular, for the three reviewers, the acknowledgment of areas of uncertainty and reference to additional sources of information was present only in 18.9% and 41.5% of the cases, respectively. The authors recognized a board certification in only 12.3% of the cases. The evaluations of the three reviewers demonstrated a strong and significant correlation (Cronbach α = 0.911; *p* < 0.001) [Figure 2].

The median MD score of the three reviewers did not present any significant correlations with the number of views (r_p_ = −0.032, *p* = 0.743), duration (r_p_ = 0.181, *p* = 0.064), interaction index (r_p_ = 0.072, *p* = 0.467), viewing rate (r_p_ = 0.014, *p* = 0.886), continent of origin (χ^2^ = 4.13, *p* = 0.248), or presence of animations (F = 0.082, *p* = 0.776).

### 3.3. The Global Quality Scale Results

The mean GQS score was 2.987 ± 1.012 for the three professional reviewers and 3.469 ± 0.891 for the three lay reviewers. The differences between the median values of the two groups evaluated with the Wilcoxon signed rank test were statistically significant (professional reviewers 3 (IQR 1.33) versus lay reviewers 3.33 (IQR 1); *p* < 0.001). The correlations between the ratings were significant between the reviewers within the same group but not between the two groups (Table 4).

In the professional group, the median GQS score of the three reviewers did not present any significant correlations with the number of views (r_p_ = −0.104, *p* = 0.287), interaction index (r_p_ = 0.089, *p* = 0.364), viewing rate (r_p_ = −0.023, *p* = 0.817) or continent of origin (χ^2^ = 4.250, *p* = 0.236). The correlation with the video duration was significant but weak (r_p_ = 0.261, *p* = 0.007). Similarly, the median GQS score of the three lay reviewers did not present any significant correlations with the number of views (r_p_ = 0.101, *p* = 0.303), duration (r_p_ = 0.100, *p* = 0.309), interaction index (r_p_ = 0.044, *p* = 0.656), viewing rate (r_p_ = −0.0005, *p* = 0.959) or continent of origin (χ^2^ = 0.877, *p* = 0.831).

**Figure 3:**
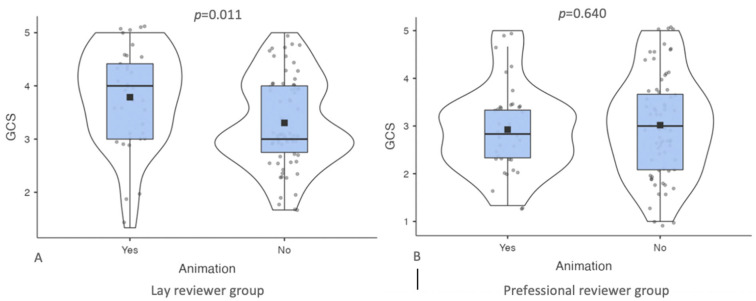
The GCS median differences between the videos with and without animations in the lay and professional reviewer groups.

The presence of animations significantly influenced the GQS score expressed by the lay reviewers (animation 4 (IQR 1.42) versus no animation 3 (IQR 1.25); *p* = 0.011) but not that of the professionals (animation 2.83 (IQR 1) versus no animation 3 (IQR 1.58); *p* = 0.640) (see Figure 3).

## 4. Discussion

YouTube and other social media have acquired a central role in people’s lives. Every day, all over the world, online video platforms are used to acquire and exchange information of all kinds, including health information [23,42]. In particular, patients with chronic debilitating pathologies and pathologies with poorly established therapeutic pathways and, frequently, unsatisfactory results, such as TMJD, use these tools to share their experiences and seek answers to their problems [43,44,45]. This profoundly affects patients’ expectations and the trust that they place in the professional who treats them [26,46,47].

In agreement with the results found in relation to many other pathologies and treatments, the quality of YouTube videos about TMJDs was on average poor [27,28,29,30,31,34,36,37,38,39]. Notably, fewer than 15% of the videos featured a clear disclosure of advertising and financial interests. The content of the videos should therefore be interpreted with caution because the purpose of the video producer could be to recruit new patients rather than actually to inform them. This content is supported by solid and explicit bibliographic sources only in one-third of the cases. In relation to most of the videos, it is therefore not possible to establish the level of evidence of the information which, considering that only 12.3% of the authors were board-certified professionals, cannot even be considered as presenting an expert opinion. Any areas of uncertainty with respect to the topic are only acknowledged in fewer than 20% of the videos analyzed. In a pathology as complex as TMJD, this could lead patients to overestimate the effectiveness of some treatments or could create expectations that cannot be met.

The evaluations expressed by the professional reviewers presented a strong and significant correlation, the reviewers assigning an overall mediocre judgment to the quality of the information present in the videos. To the best of our knowledge, this is the only published study evaluating the quality of health information on YouTube which has included an evaluation by a lay audience. The reviewers in this group rated the videos only with the GQS: a simple tool which is limited to a subjective evaluation of the quality and flow of information [33]. The MD and HONcode involve an assessment of some characteristics of the quality of health information, which means that any reviewer requires superior scientific training in order to give a reliable judgment [48]. In other words, the lay reviewer would not have the expertise to establish whether a content creator has a board-certification, whether the information presented is balanced and unbiased or whether areas of uncertainty have been acknowledged [49]. On the other hand, an analysis of public opinion is essential for an understanding of which characteristics can capture the attention of users to allow for a maximum diffusion of quality content. In this study, the GQS scores of the three lay reviewers were strongly correlated. Overall, their ratings were significantly higher than those expressed by the professional reviewers. Furthermore, the GQS scores of the lay group were significantly higher when there were animations included in the evaluated videos. Animations can be useful in order to make the message conveyed by the video more easily understandable [50,51], especially considering the complex pathophysiology of TMJD, which is often little known even by healthcare professionals [19].

The findings of this study highlight that most of the information on YouTube about TMJD is not created by board-certified specialists. This should prompt academic healthcare institutions and professional organizations to fill this quality gap by creating content that is scientifically accurate and at the same time manages to capture the attention of patients. Furthermore, online platforms should implement health information quality control systems which must not be limited to the number of likes given by the lay public but should be based on some form of peer review, such as that of scientific journals. On the other hand, the treatment of TMJD involves a step therapy. Although the steps are burdened by a significant failure rate, it is necessary to go through each one before moving onto the next. Professionals dealing with the treatment of TMJD must strive to build a deep relationship of trust with their patients based on quality counseling to prevent them from resorting to unreliable information found on the internet after a first therapeutic failure. Finally, health systems should implement programs to educate people in the prudent interpretation of the health information available online by providing them with tools which will enable them to judge critically its quality based on the parameters commonly used by the scientific community

This study has certain limitations. First, only videos in English and with more than 10,000 views were included, meaning that it is possible that there are other higher quality videos, either produced in other languages or more recently published, which were not taken into consideration. However, there is a justification for the adoption of this strategy in that we wanted to include only the videos with the widest circulation. Secondly, the content of the platform is rapidly and continuously evolving, and it is possible that the information available may have changed significantly within a short time period. Thirdly, the results are based on the judgment of a small sample of professionals and lay people who may not constitute a completely reliable and representative expression of a wider judgment.

## 5. Conclusions

The quality of information on TMJD on YouTube is generally of poor quality. Healthcare systems and professionals should be prepared to correct such misinformation and should aim to build trusting relationships with patients which are based on quality counseling. Similarly, academic institutions should produce quality content that guides patients with TMJD toward a correct diagnostic-therapeutic process.

## Figures and Tables

**Table 1:** The Health on the Net Foundation code of conduct tool (HONcode).

Principle	Definition
Authoritativeness	Indicates the qualifications of the authors
Complementarity	The information supports, rather than replaces, the doctor–patient relationship
Privacy	Respects the privacy and confidentiality of any personal data submitted to the site by the visitor
Attribution	Cites the sources of the published information, data, and medical and health pages
Justifiability	Supports with evidence any claims relating to benefits and performance
Transparency	Presents accessible and accurate email contacts
Financial disclosure	Identifies funding sources
Advertising policy	Clearly distinguishes advertising from editorial content

**Table 2:** Modified DISCERN (MD).

Video Characteristics
1. Are the aims clear and achieved?
2. Is reliable information used (i.e., are publications cited? Is the speaker a board certified practitioner?)?
3. Is the information presented balanced and unbiased?
4. Are additional sources of information listed for patient reference?
5. Are areas of uncertainty mentioned?

**Table 3:** The Global Quality Scale (GQS).

Score	Definition
1	Poor quality, poor flow of the video, most important information missing, not useful for the patient
2	Generally poor quality and poor flow of the video, some significant information included but many important topics missing, of very limited use to the patient
3	Moderate quality, suboptimal flow, some important information is adequately presented but other topics are poorly discussed, somewhat useful for the patient
4	Good quality and generally good flow, most of the relevant information is included but some topics are not covered, useful for the patient
5	Excellent quality and flow, very useful for the patient

**Table 4:** Correlation analysis between the GQS scores of the six reviewers.

		GQS Score Lay Reviewer 1	GQS Score Lay Reviewer 2	GQS Score Lay Reviewer 3	GQS Score Professional Reviewer 1	GQS Score Professional Reviewer 2	GQS Score Professional Reviewer 3
GQS score lay reviewer 1	Pearson’s r	-					
	*p*-value	-					
GQS score lay reviewer 2	Pearson’s r	0.697	-				
	*p*-value	<0.001	-				
GQS score lay reviewer 3	Pearson’s r	0.743	0.871	-			
	*p*-value	<0.001	<0.001	-			
GQS score professional reviewer 1	Pearson’s r	0.016	−0.015	−0.094	-		
	*p*-value	0.868	0.881	0.335	-		
GQS score professional reviewer 2	Pearson’s r	0.112	0.092	−0.008	0.774	-	
	*p*-value	0.252	0.349	0.937	<0.001	-	
GQS score professional reviewer 3	Pearson’s r	0.157	0.167	0.086	0.685	0.783	-
	*p*-value	0.108	0.087	0.381	<0.001	<0.001	-

## Data Availability

L.A.V., S.S. and G.D.R have full access to the data reported in this study.
